# Wearing American Football helmets increases cervicocephalic kinaesthetic awareness in “elite” American Football players but not controls

**DOI:** 10.1186/s12998-015-0077-4

**Published:** 2015-11-16

**Authors:** Peter W. McCarthy, Phillip J. Hume, Andrew I. Heusch, Sally D. Lark

**Affiliations:** Faculty of Life Sciences and Education, University of South Wales, Pontypridd, Mid-Glamorgan, Wales CF37 1DL UK; Anglo-European College of Chiropractic, Parkwood Road, Bournemouth, Dorset BH5 2DF UK; Massey University Wellington, College of Health, P O Box 756, Wellington, 6140 New Zealand

**Keywords:** Neck function, Proprioception, Sports, Protective equipment

## Abstract

**Background:**

While there have been investigations into the reduced neck injury rate of wearing protective helmets, there is little information on its effects on normal kinaesthetic neck function. This study aims to quantify the kinaesthetic and movement effects of the American football helmet.

**Methods:**

Fifteen British Collegiate American football players (mean age 22.2, SD 1.9; BMI kg.m^2^ 26.3, SD 3.7) were age and size matched to 11 non-American football playing university students (mean age 22.5, SD 3.6; BMI 24.3, SD 3.3 kg.m^2^). Both groups had their active cervical range of motion and head repositioning accuracy measured during neck flexion/extension using a modified cervical range of motion device and a similarly modified football helmet.

**Results:**

Wearing helmets significantly reduced active cervical range of motion in extension in both groups (*P* = 0.007 and *P* = 0.001 Controls and American Footballers respectively). While both groups had similar repositioning when not wearing a helmet (flexion *P* = 0.99; extension *P* = 0.52), when wearing helmets, American football players appeared to be more accurate in relation to cervical kinaesthetic repositioning (ANOVA: *P* = 0.077: flexion effect size =0.84; extension effect size =0.38).

**Conclusions:**

Wearing American football helmets significantly reduces the active cervical range of motion in extension, along with a change in the neutral head position. American footballers have a greater accuracy in repositioning their head from flexion (potentially enhanced proprioception) when wearing a helmet. This finding might allow development of a simple objective test to help discern presence of minor concussive or cervical musculoskeletal injury on or off the field.

## Background

Afferent proprioceptive information is important for sensorimotor control of posture and movement [1]. Joint disease and other musculoskeletal conditions can associate with altered proprioceptive functioning [2–4] which, in extreme cases such as following joint injection in the neck can result in ataxia; ipsilateral hypotonia of the arm and leg and a strong sensation of falling or tilting [1]. Although extreme, this highlights the potential for lesser changes in neural feedback to affect the fine motor control crucial for elite performance.

American Football (AF) is the third highest source of sports injuries being responsible for over one million reported injuries a year within the United States alone [5]. Concussion appears the most common injury type, with 17.78–26.95 concussive and neck neurologic injuries per 10,000 athlete exposures [6]. Injuries to the cervical spine are the most common catastrophic injuries in AF [7], and the second highest cause of death within the sport over the period 1977–2001 [8]. Consequently strict rules and considerable protective clothing have reduced the severity of impacts to the head and body [7, 9, 10]. Cumulative effects of more frequent lesser neuro-musculoskeletal trauma (e.g., minor head injury) tend to be ignored in contact sports that require repetitive short bursts of maximal effort (American Football and Rugby football).

Embedding use of protective equipment into training and gameplay helps familiarization and adaptation. However, equipment may have predictable additional effects (visual and auditory impairment) and less immediately apparent postural adaptations, decreased cervical spine function (range of motion [ROM] and cervicocephalic kinaesthetic repositioning: [CKR]). In elite professional Rugby football players cervical spine range of motion appears related to both game play and time in the sport [11, 12]. In apparent contrast, AF players [13] have a greater active cervical range of motion (ACROM). However, to the author’s knowledge, there is no information available concerning the possible consequences of wearing a protective helmet in terms of its’ added mass, displaced centre of gravity and neutral head position and CKR.

There is evidence of deficits in CKR (interchangeable with the term proprioceptive deficits) resulting from trauma, such as in whiplash [14]; however, Rugby players also had a significantly decreased ability to reposition the head to a neutral position following neck extension [11] or rotation [15]. If the helmet is truly protective, AF players, who are subject to similar forces (impact and shear forces) to Rugby, should not have the same degree of deficit in CKR seen in the Rugby player [11, 12, 16].

The aim of this study was to determine whether the wearing of protective headgear by AF players influences active range of motion in the neck and CKR as assessed by head repositioning.

## Method

The population size for this study was determined from previous studies of active cervical range of motion (ACROM and CKR effect sizes 0.3 to 1.2) [11]. Fifteen AF player volunteers (22.2 SD 1.9 years) were recruited from the British Collegiate American Football League. All players came from one of the national semi-finalist teams (Cardiff University Cobras, Southampton University Stags England), with nine volunteers having represented Great Britain at the collegiate level (equates to playing in the National Collegiate Athletic Association [NCAA] Division III within the USA). For inclusion in this study each player had to have had a minimum of 3 years playing experience of full contact, kitted AF. Further criteria included participants being currently asymptomatic for neck pain or discomfort. A screening questionnaire was completed by all recruited participants to determine presence of current or previous neck trauma, surgery or disorders that may exclude them from participating in the study or influence the results: e.g., dizziness, tinnitus, diabetes mellitus, asthma, hypertension, headaches/migraines.

Initially 15 age and size matched control volunteers were recruited, however only 11 of these fulfilled the inclusion criteria (*n* = 11; 22.5 SD 3.6 years). Controls were trained athletes who participated in non-contact amateur competitive sports such as triathlon, swimming, water polo and basketball. All participants volunteered and gave written informed consent after receiving verbal and written information about the study, which was approved by the ethics committee of the School of Applied Sciences, University of Glamorgan, and follows the Helsinki Declaration ethical guidelines.

The method employed here is based on that described previously [11]. The protocol will be described in 3 sections: anthropometrics, assessment with a cervical range of motion (CROM) device, and helmeted assessment. The study presentation order regarding CROM or helmet measurements was randomised between participants to remove potential order effects.

**Fig. 1:**
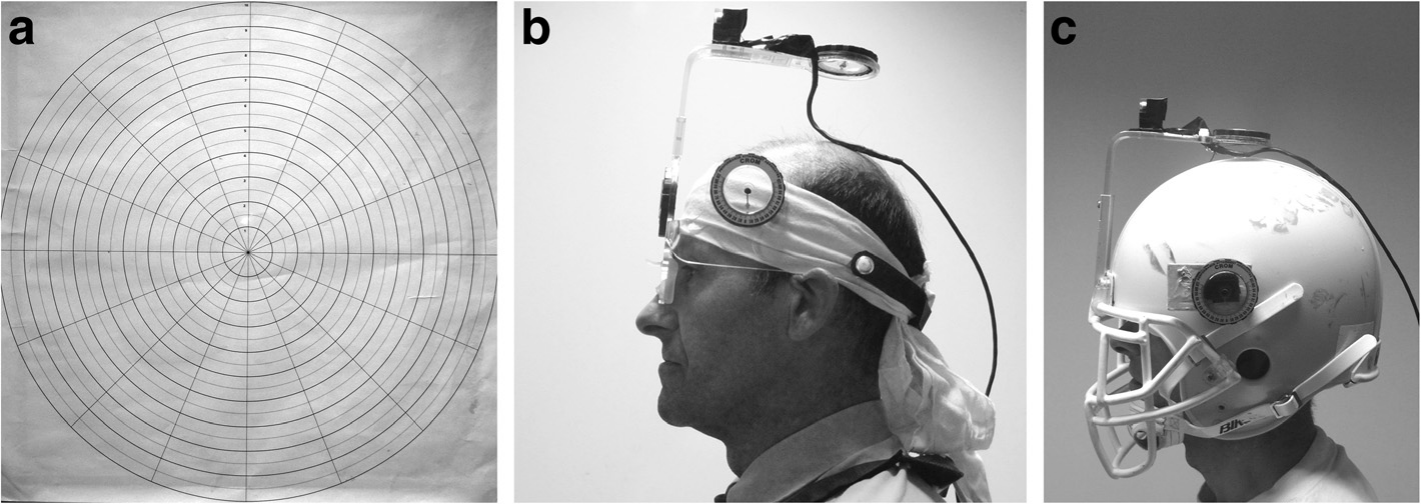
This figure shows the measurement chart target for quantifying repositioning error (**a**) and the cervical range of motion (CROM) devices used for assessing unhelmeted (**b**) and helmeted (**c**) range of motion. Figure 1a shows the wall chart *in situ* illustrating the concentric evenly placed circles about the (0, 0) centre point with a laser light visible between rings labelled 1 and 2 above the centre spot. In Fig. 1b, the modified CROM can be seen showing the side mounted gravity goniometer and the rotational arm suspended above vertex of the subject’s head which holds the mounted pencil laser. The final element (1c) shows the modified American football helmet with the rotational arm of the CROM mounted on the front of the helmet and gravity goniometer on the side in line with the vertex of the helmet. In all cases, the rotational arm of the goniometer was only used as a stable point to mount the pencil laser

Anthropometric measures: neck girth (Hoechstmass HM-82203 Rollfix Tape Measure; Cranlea, Bourneville UK), body mass (floor scales model 761, Seca GmBH, Germany) and height (stadiometer model 202, Seca GmBH, Germany) were recorded. Participants sat in a chair which was height adjustable, so that their hips, knees and ankle angles were all set to ~90° SD 5°, as assessed by a professional quality JAMAR E-Z Read goniometer (Physiomed, Manchester, UK). The chair was positioned so that the vertex of the participants’ head was 1 m (SD 5 %) from a custom made wall mounted chart (Fig. 1a) that was to receive light from a laser either mounted onto the CROM device (Performance Attainment Associates, Lindstrom MN, USA) or AF helmet with face guard (see below and Fig. 1b & c for details). The participants were instructed not to arch their thoraco-lumbar spine during extension, to ensure only neck muscles were engaged in the flexion-extension movements. A biofeedback cuff (Stabilizer™ pressure bio-feedback, Chattanooga group, Encore Medical Texas, USA) was placed between the posterior upper thoracic spine region and the back of the chair and inflated to 40 mm Hg. Measurements were repeated if the pressure deviated by >2 mm Hg during head movements. Each participant was guided through a warm-up and familiarization session (the equivalent of three repeated trials) before assessment began.

To measure ACROM, a cotton bandana was tied around the head (above the brow anteriorly and tied posteriorly above the occiput), to cushion the body of a CROM device and ensure stability regardless of idiosyncrasy associated with head/skull morphology. The CROM was placed onto the head as described by the manufacturers: the magnetic yolk was not used in these experiments, as rotation was not measured in this study. CROM devices have been used extensively in this type of research and have been shown to have sufficient accuracy [17], validity [18, 19], and reliability [20] for studies of ACROM such as this. This CROM device had a custom made laser block (Perspex block containing a pencil laser (class 3a: 650 nm: miraclebeam™ Pacoima, CA) mounted (screwed) on the rotational arm slightly forward of the position which would overly the vertex (Fig. 1b). This was used for assessment of CKR using the CROM or adapted helmet.

The participants’ ACROM in full flexion and full extension was assessed as follows: the participant was asked to maximally flex their head forwards by tucking in their chin to their chest, or extend their head back while maintaining their shoulders and mid-to lower back in a normal upright position (including their normal curvature). There was a 2 s hold at the end of each movement to establish the end point reading (angle). After each head movement, the participants were asked to return to their neutral starting position (looking directly ahead).

To assess ACROM wearing the football helmet a standard mid-sized AF helmet and grill was adapted as follows (Fig. 1c): an attachment for the rotational arm of the CROM was custom made of aluminium and bolted onto anterior midline of the helmet; between the two upper anterior grill anchor points. A further custom-made aluminium block was bolted on the left lateral aspect above the grill attachment point (in line with the vertex in the coronal plane). This was used to affix a gravity goniometer that had been extracted from a spare CROM device. Using the same type and manufacture of goniometer allowed the modified helmet assessment to have comparable reading accuracy to the CROM.

Laser repositioning was used in the assessment of CKR. Participants were asked to close their eyes and find a comfortable neutral head position, at which point the laser was switched on. Once the laser light was visible on the wall mounted chart (Fig. 1a), the chart was moved so that the laser light impacted the centre of the chart (position 0, 0). Following chart alignment, participants were instructed to repeat the flexion and extension movements (returning to their perceived neutral position between movements) keeping their eyes closed. Repositioning was assessed by returning to perceived neutral from both full flexion and full extension, with the order of head movement alternated to reduce any order effect. The CKR was recorded using an adaptation of the procedure reported by Revel [21]. Once the participant affirmed they had returned to neutral, the actual position of the laser on the wall chart was noted (distance from the centre, direction in relation to undershoot or overshoot as well as lateral deviation).

Data was tested for skewness and kurtosis and deemed to be normally distributed for statistical analysis. A repeated measures ANOVA was used to identify main effects, post-hoc analysis using the Paired Student’s *T*-test for the helmet effect separately in each group (controls and AF). As direction of change was not immediately predictable, 2-tailed analysis was used. Probability values of 0.1 to 0.05 were considered as signifying strong trends and values <0.05 were deemed a significant change. Effect size (ES) was calculated using the method of Cohen’s D [22]. Statistical analysis was performed using SPSS 18.0 for Windows.

## Results

**Fig. 2:**
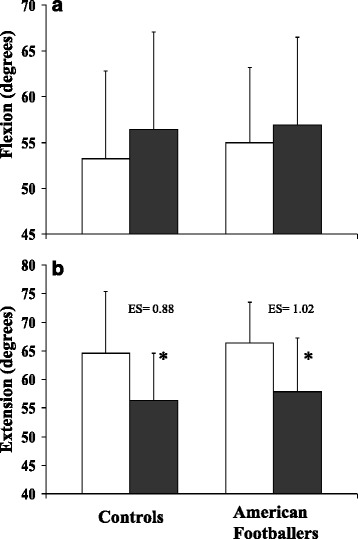
Mean ± SD values for cervical range of motion in American football players and controls subjects in flexion (**a**) and extension (**b**) while either wearing a football helmet (*blocked bars*), or not (*unblocked bars*). * denotes a significant difference (*P* < 0.05) between wearing a helmet or not. Effect sizes (ES) are shown as helmet vs no helmet for each group

Age and anthropometric characteristics of the participants are shown in Table 1. Although the AF players appeared heavier than controls, this was not statistically significant instead showing a strong trend (*P* = 0.085). Both groups of participants had reportedly sustained similar numbers of concussive injuries overall and had similar ACROM in flexion and extension (*t*-test: *P* = 0.62 and *P* = 0.63 respectively). The main ACROM effects calculated from the repeated measures ANOVA reveal that wearing a helmet affects the ACROM (*P* = 0.014) with no difference between the controls and AF players (*P* = 0.62). Although wearing a helmet did not significantly alter the ACROM in flexion for both controls and AF players (paired t-tests: *P* = 0.14 and *P* = 0.31 respectively: Fig. 2a), it significantly decreased the ACROM in extension (paired t-tests: *P* = 0.007 [ES = 0.88] and *P* = 0.001 [ES = 1.02]; controls and AF players respectively: Fig. 2b). To determine whether the changes in ACROM were the result of a displacement in the neutral point, the ratio between flexion and extension (flexion/extension) was calculated (Table 2).

**Table 1:** Anthropometric and Concussive Injury Measures

	Age (years)	Height (m)	Mass (kg)	BMI	Neck Girth (cm)	Concussive Head Injuries
Control (*n* = 11)	22.5 (3.6)	1.77 (9.6)	76.2 (11.9)	24.2 (3.3)	39.1 (2.3)	1.3 (0.5)
American Football players (*n* = 15)	22.2 (1.9)	1.81 (7.5)	87.1 (17.3)	26.3 (3.7)	39.0 (2.4)	1.5 (0.5)

Mean (±1 standard deviation) for the anthropometric measures and the number of concussion head injuries declared for Controls and American Football players

**Table 2:** Flexion-Extension Ratios

		Helmet	No Helmet
Controls (*n* = 11)	range	0.75–1.39	0.61–1.15
	average	1.01 ± 0.21	0.85 ± 0.20
AF Players (*n* = 15)	range	0.57–1.62	0.59–1.30
	average	1.04 ± 0.21	0.87 ± 0.18

Ratios of the active cervical range of motion (ACROM) for amount of flexion compared to extension can be used as an indication of the subject’s preferred neutral point under those conditions. Both groups were measured with and without an American footballers (AF) helmet. Both AF and control groups significantly changed their flexion-extension ratio whilst wearing the helmet (*p* < 0.01). Data is presented as mean ± 1 standard deviation; however the range is also presented to allow greater clarity regarding the changes seen

**Fig. 3:**
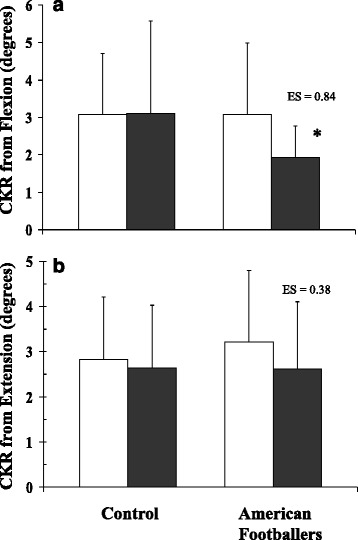
Mean ± SD values for cervical kinaesthetic repositioning (CKR) to a self-selected neutral point from flexion (**a**) or extension (**b**). The histograms shows the amount of variation (degrees) on return to a self-selected neutral head position for American football players and control participants while either wearing a football helmet (*blocked bars*), or not (*unblocked bars*). * denotes significant difference (*P* < 0.05) between American footballer players with helmets on and all other groups: flexion (**a**) only. Effect sizes (ES) relate to helmet vs no helmet for the American Football group

Generally, AF players had similar CK repositioning to the controls when not wearing a helmet (unpaired t-tests: returning from flexion *P* = 0.99, or extension *P* = 0.52). Wearing the helmet significantly enhanced the CK repositioning ability (ANOVA: *P* = 0.04: Fig. 3a and b). However, AF players appeared to be more accurate than the controls in relation to CK when wearing the helmet (ANOVA: *P* = 0.077). The CK of the control participants appeared unaffected either returning from flexion (paired *t*-test: *P* = 0.93) or extension (paired *t*-test: *P* = 0.6). In contrast, wearing helmets enhanced the AF players CK: flexion with no helmet 3.1°, with helmet 1.9°, (paired *t*-test: *P* = 0.054: [ES = 0.84] Fig. 3a); extension with no helmet 3.2°, with helmet 2.6° (paired *t*-test: *P* = 0.22: [ES = 0.38] Fig. 3b). Direct comparison between controls and players showed that when returning from flexion, helmeted players had better CK than helmeted controls (ES = 0.71). All other comparisons had ES <0.20.

## Discussion

Wearing an AF helmet appears to have affected the ACROM of the user regardless of group and the CK repositioning error to the benefit of the trained wearer. ACROM assessment revealed similar effects in both groups with the most noticeable being a significant decrease in extension. From a physical perspective, participants in this study were generally not significantly different between groups (Table 1). Additionally, neck girths were of similar circumference in both groups and were within the range reported [23] for young AF players; however, there was no neck circumference data available from older AF players for comparison.

A possible explanation for the decrease in extension and lack of significant change in flexion could be a re-alignment of the head so that the neutral point is further into extension (Table 2) coupled with the physical restriction associated with wearing the helmet. If the change in flexion was equal and opposite to that in extension, one could surmise that this was due to neutral point deviation alone. However as the change was not equal and opposite, the additional difference could result from a physical restriction to movement caused by the helmet. The slight difference between Pearl and Mayer (1979) [23] and the results presented here regarding changes in flexion ACROM, tend to support this hypothesis: although, the presence of shoulder pads worn in the Pearl and Mayer [23] study could be considered to have contributed to an additional reduction in flexion range of motion. The effect of the helmet’s mass appeared to have resulted in realignment of the head’s neutral position on the cervical spine towards extension. Thereby decreasing available range for extension and increasing that for flexion, as can be seen in the significant change in flexion-extension ratio (Table 2). Interestingly, the reset neutral point became equidistant for flexion and extension, whereas without the helmet the ratio was in favour of flexion. Such a change in muscular balance over time might be expected to result in hypertonicity of the neck musculature which in turn might restrict return to the unhelmeted position; however, there was no apparent evidence for this in the data. Adaptations in either muscle length or tonicity related to the helmeted neutral position do not appear to have occurred in these younger AF players; as their unhelmeted and helmeted ACROM results are almost identical to the controls. It would be interesting to determine if older or elite professional AF players maintain this characteristic or show more permanent adaptation to helmet wear, in which case use of electromyography might help determine if increased muscle activity is involved in any change.

Although it was considered that the AF players might gain advantage from the prolonged use of the helmet in training and play, it was surprising to find that the controls did not suffer from a reduced repositioning accuracy when put into the unfamiliar situation of wearing the helmet (Fig. 3a and b). In addition to simply wearing the helmet, the adaptations made by the participant, such as a potential displacement of the neutral position (flexion:extension ratio; Table 2), would be expected to exacerbate potential for reduced repositioning accuracy. The *significantly* lower error in repositioning from flexion *(from 3.08° to 1.93°)* for the AF player population when wearing the helmet (*P* = 0.054: ES = 0.84) suggests that the regular wearing of the helmet can create an advantage in repositioning accuracy. The exact cause of this is unknown, but could include: mass of the helmet, displacement of the centre of gravity, and/or the specific training and repetitive use with some element of feedback or “reward” affecting neurological programming. While integration of all the afferent information is at a higher level of the vestibular nuclei, the vestibular system reflexes are closely coupled to cervicospinal reflexes and activation of the neck muscles increases vestibular responsiveness via the combined cervico-collic and vestibulo-collic reflexes. It has been proposed that sustained cervico-spinal reflex activation affords a prolonged after-effect to enhance the vestibulo-collic reflex (Pettorossi and Schieppati 2014) [24]. This would equate to the habituation of increased loading on the head and neck muscles by the helmet. Lack of equivalent change in the control group supports this hypothesis and suggests that incorporating useful feedback from wearing of the helmet might require a training period. The lack of difference between the AF players and the controls prior to putting on the helmet supports this conclusion. It might be worth considering whether there are general benefits conferred by the enhanced CK repositioning to a self-selected neutral position when wearing the helmet. However, this was not apparent in the AF players available for this study when returning from extension (from 3.21° to 2.62°: *P* = 0.22: ES = 0.38). It is possible that a different result might be found with players of a higher standard (i.e. higher than National Collegiate Athletic Association Division III). Furthermore, there are a number of additional questions raised by these results: such as whether position of play has any specific relationship to changes in repositioning accuracy?

Although the effects of the helmet were tested without the participants being in full kit, a number of points can still be drawn from the results of the study. Primarily, wearing the helmet affects the flexion:extension ratio and reduces total available ACROM. Although this would be expected to cause adaptations in neck use for the player, these were not apparent in these younger players, but muscle length and strength changes along with associated cervical spine joint damage might accrue chronically so become apparent in older players. It has been well documented that such changes can result in symptoms such as headache [25]. However, although headache is very common in AF as a result of direct head contact [26], resolving the effects of adaptations in the neck muscles to the helmet alone will probably be too subtle or inconsequential to be determined by this method.

The enhanced CKR might have implications in the detection of neurological and musculoskeletal impairment in AF such as following minor concussion and or recovery from neck trauma [27]. Detection of minor concussion or determining full recovery are recognised problems in AF. Most methods employable during a game are limited and usually test for gross neurological compromise such as gross disturbance of proprioception, (standing and walking tests), which tend to miss the more subtle changes, making accurate determination of recovery difficult for the clinician. Furthermore, subjective tests such as those for pain tend to be hidden when elite players wish to remain on the field. There is evidence to support a relationship between presence of subclinical pain and changed cervicocephalic kinaesthetic sensibility [28] which strengthens the possible usefulness of a tool to objectify neurological damage on the field. Finding an objective tool to help determine level of neurological or musculoskeletal damage following a collision is important when taken into context with the additive effect of further head collisions which have more profound implications to outcome [29]. However, as fine neurological processing skills including CKR, can be easily lost following a concussive injury [30] or musculoskeletal damage akin to whiplash, the use of a simple testing system such as this, on a fully kitted player, might allow future development of a more reliable field based test for the presence of such damage following head collision in elite sport.

## Conclusions

In conclusion wearing an AF helmet causes a significant reduction in extension ACROM in AF players and controls, as well as potentially disturbing the flexion:extension ratio. Constantly wearing a helmet results in AF players developing improved CK when returning from full flexion but not extension, short term wearing does not give any beneficial effects in terms of CK.

There is a need to determine the cause and extent of this improvement, also to determine whether player position/role and standard of play affects the size and direction of change.

### Practical implications

- Applications of this research might help develop a sensitive proprioceptive test for AF players suffering from a concussive injury, which can be applied without removing the helmet.
- This research might be extrapolated to other helmet wearing sports and occupations.
- Future study of AF players might give an insight into enhancing proprioceptive skill acquisition in sport.

## Abbreviations

ACROM: Active cervical range of motion
AF: American Football
CROM: Cervical range of motion
CKR: Cervicocephalic kinaesthetic repositioning
ROM: Range of motion
ES: Effect size
